# High Altitude Increases Alteration in Maximal Torque but Not in Rapid Torque Development in Knee Extensors after Repeated Treadmill Sprinting

**DOI:** 10.3389/fphys.2016.00097

**Published:** 2016-03-14

**Authors:** Olivier Girard, Franck Brocherie, Grégoire P. Millet

**Affiliations:** ^1^ISSUL, Institute of Sport Sciences, Faculty of Biology and Medicine, University of LausanneLausanne, Switzerland; ^2^Athlete Health and Performance Research Centre, Orthopaedic and Sports Medicine HospitalAspetar, Doha, Qatar

**Keywords:** repeated-sprint ability, hypoxia, rapid torque development, neural drive, voluntary force production

## Abstract

We assessed knee extensor neuromuscular adjustments following repeated treadmill sprints in different normobaric hypoxia conditions, with special reference to rapid muscle torque production capacity. Thirteen team- and racquet-sport athletes undertook 8 × 5-s “all-out” sprints (passive recovery = 25 s) on a non-motorized treadmill in normoxia (NM; FiO_2_ = 20.9%), at low (LA; FiO_2_ = 16.8%) and high (HA; FiO_2_ = 13.3%) normobaric hypoxia (simulated altitudes of ~1800 m and ~3600 m, respectively). Explosive (~1 s; “fast” instruction) and maximal (~5 s; “hard” instruction) voluntary isometric contractions (MVC) of the knee extensors (KE), with concurrent electromyographic (EMG) activity recordings of the *vastus lateralis* (VL) and *rectus femoris* (RF) muscles, were performed before and 1-min post-exercise. Rate of torque development (RTD) and EMG (i.e., Root Mean Square or RMS) rise from 0 to 30, −50, −100, and −200 ms were recorded, and were also normalized to maximal torque and EMG values, respectively. Distance covered during the first 5-s sprint was similar (*P* > 0.05) in all conditions. A larger (*P* < 0.05) sprint decrement score and a shorter (*P* < 0.05) cumulated distance covered over the eight sprints occurred in HA (−8 ± 4% and 178 ± 11 m) but not in LA (−7 ± 3% and 181 ± 10 m) compared to NM (−5 ± 2% and 183 ± 9 m). Compared to NM (−9 ± 7%), a larger (*P* < 0.05) reduction in MVC torque occurred post-exercise in HA (−14 ± 9%) but not in LA (-12 ± 7%), with no difference between NM and LA (*P* > 0.05). Irrespectively of condition (*P* > 0.05), peak RTD (−6 ± 11%; *P* < 0.05), and normalized peak RMS activity for VL (−8 ± 11%; *P* = 0.07) and RF (−14 ± 11%; *P* < 0.01) muscles were reduced post-exercise, whereas reductions (*P* < 0.05) in absolute RTD occurred within the 0–100 (−8 ± 9%) and 0–200 ms (−10 ± 8%) epochs after contraction onset. After normalization to MVC torque, there was no difference in RTD values. Additionally, the EMG rise for VL muscle was similar (*P* > 0.05), whereas it increased (*P* < 0.05) for RF muscle during all epochs post-exercise, independently of the conditions. In summary, alteration in repeated-sprint ability and post-exercise MVC decrease were greater at high altitude than in normoxia or at low altitude. However, the post-exercise alterations in RTD were similar between normoxia and low-to-high hypoxia.

## Introduction

Intense physical efforts performed at or near maximal speed and the ability to recover from it are important markers of successful in-game performance in high-intensity, intermittent sports (Spencer et al., 2005). Team- and racquet-sport players implement training methods, based on the repetition of maximal efforts in normoxia (e.g., repeated sprints, Bishop et al., 2011) or in hypoxia (e.g., repeated sprints in hypoxia; Brocherie et al., 2015a), for eliciting neuromuscular adaptations (e.g., enhanced muscle oxygenation and activation responses) and improving cardiovascular and metabolic function, in turn maximizing their physical performance. Although the physiological responses and potential metabolic limiting factors (i.e., limitations in energy supply, metabolite accumulation) associated with the completion of one repeated-sprint set have largely been described, there is comparatively less data on the neuromuscular consequences (Girard et al., 2011).

Neuromuscular fatigue is an exercise-induced reduction in the maximal isometric voluntary contraction (MVC) force/torque or power of a muscle group, which potentially involves alterations at any levels from the brain to skeletal muscles (Gandevia, 2001). Over the past decade, an increasing number of studies have quantified neuromuscular fatigue following repeated running (Perrey et al., 2010) or cycling (Racinais et al., 2007; Billaut et al., 2013; Girard et al., 2013; Hureau et al., 2014) sprints by comparing pre- to post-sprint values of force/torque, voluntary activation, electromyogram (EMG), and twitch responses. Recent studies assessed the neuromuscular function during the actual repeated-sprint sets (Goodall et al., 2015; Pearcey et al., 2015). With regard to MVC, however, it is important to note that maximal force/torque production capacity is most often obtained when exceeding 300 ms following contraction onset (Thorstensson et al., 1976). This is contrasting to the characteristics of muscular contraction occurring in several sporting events, where muscle force needs to be developed in less than 250 ms (e.g., sprints: Kuitunen et al., 2002; jumps: Luhtanen and Komi, 1979). Therefore, the ability to rapidly generate force/torque, i.e., the rate of force/torque development (RTD) within the initial (i.e., <250 ms) phase of an MVC which in turn correlates with sprint performance (Tillin et al., 2013), likely constitutes a more functional outcome measure (Girard and Millet, 2009). Therefore, RTD as a surrogate for explosive strength should be assessed for a better understanding of the acute neuromuscular adjustments to repeated sprinting.

Repeated-sprint ability is more altered at high (>3000–3500 m or FiO_2_ below 13–14%) than lower altitudes, either normoxia or low-to-moderate (<3000 m or FiO_2_ above 14%) altitude (Bowtell et al., 2014; Goods et al., 2014). Not only acute hypoxic exposure decreases convective O_2_ transport (i.e., reduction in arterial O_2_ saturation values or SpO_2_), but also challenges multiple regulatory systems by increasing cardiorespiratory (i.e., higher heart rate, minute ventilation, O_2_ debt), metabolic (i.e., slower muscle re-oxygenation responses) and/or neuromuscular (i.e., incomplete muscle activation) requirements during sprinting or subsequent recovery periods (Balsom et al., 1994; Billaut et al., 2013; Bowtell et al., 2014). Compared to normoxia, the completion of a repeated-sprint cycling protocol (15 × 5-s efforts, 25-s rest) in high hypoxia (FiO_2_ = 14%) led to ~8% lower total mechanical work as a result of impaired muscle activation, which was also accompanied by ~6% lower post-exercise MVC (Billaut et al., 2013). However, these authors did not include any measure of explosive strength in their study.

To our knowledge, only one study has investigated the effect of repeated sprinting (i.e., 10 × 6-s “all out” cycling sprints, followed, after 6 min of passive rest, by 5 × 6-s sprints; recoveries = 30 s) performance on post-exercise alterations in rapid muscle torque production capacity of the knee extensors (KE) (Girard et al., 2013). MVC (−12%) and RTD (−15 to −26% from the 0–30 to 0–200 ms epochs after contraction onset) decreased during brief (i.e., 5 s) contractions after (i.e., 3 min) the repeated-sprint exercise. From this report, however, it is not entirely clear to which extent differences in RTD actually resulted from maximal voluntary strength adjustments, since RTD results were not normalized to MVC torque. Hence, whereas MVC torque losses following prolonged match-play tennis accounted for the dampened RTD values (Girard et al., 2014), fatigue induced by 10 sets of voluntary maximal explosive contractions exerted a more rapid and pronounced effect (particularly during the initial 50 ms of contraction) on explosive strength than MVC torque (Buckthorpe et al., 2014). Consequently, although repeated sprinting decreases RTD (Girard et al., 2013), the question of whether RTD at early and/or late intervals is more pronouncedly impaired than MVC has not been specifically addressed.

The analysis of EMG amplitude throughout the rising force-time curve can reveal how voluntary neural drive to skeletal muscle underlies the post-exercise decreased RTD. Hence, the rate of muscle activation is related to muscle shortening velocity (Nelson, 1996), a factor directly influencing RTD (Harridge et al., 1996). Furthermore, the determinants of explosive force production appear to change throughout the rising force-time curve (Folland et al., 2014) and fatigue may differentially affect the development of force throughout the time course of an explosive contraction. After repeated sprinting, non-significant reductions in *vastus lateralis* (VL) Root Mean Square (RMS) activities have been shown to accompany deteriorated RTD values (Girard et al., 2013). However, because the delay between exercise termination and post-exercise neuromuscular testing was 3 min in the aforementioned study, any meaningful changes in the central nervous performance may have already recovered. Furthermore, it has not been investigated if the early and later RTD time intervals (and associated rate of EMG activity rise) are in fact modified differently by performing repeated-sprint in various hypoxia severity levels. This question of the alteration in explosive strength post-hypoxic exposure/training is of high practical relevance in team- and racquet-sports but remains unclear: Hence, while countermovement jump performance increased to a similar extent after repeated-sprint training in normoxia vs. hypoxia (Brocherie et al., 2015a), movement velocity and power during the execution of a force-velocity in bench-press are improved when exposed to hypobaric vs. normobaric hypoxia in reference to normoxia (Feriche et al., 2014).

The aim of this study was therefore to assess the effects of repeated sprinting in different levels of normobaric hypoxia on the alterations in RTD and neuromuscular activity of KE. Given that there is “*extraordinarily little that changes with regard to maximal force-generating capacity with acute hypoxia”* (Perrey and Rupp, 2009), it was hypothesized that the already-known decrease in repeated-sprint ability (i.e., lower fatigue resistance) under high hypoxic conditions would not be associated with more pronounced alterations in explosive force production (i.e., RTD) compared to normoxia or low hypoxia.

## Methods

### Participants

Thirteen male recreational team- (i.e., football, rugby, basketball) and racket- (i.e., tennis, squash) sport athletes (Mean ± SD: 31.2 ± 4.8 years; 178.4 ± 6.6 cm; 74.3 ± 8.2 kg) participated in the study. All participants were born and raised at <1000 m and had not traveled to elevations >1000 m in the 3 months prior to investigation. They gave their informed, written consent preceding the commencement of the experiment. Experimental protocol was conducted according to the Declaration of Helsinki for use of Human Subjects and approved by the Ethics Committee of *Shafallah Medical Genetics Center*.

### Study design

Elements have previously been reported in Girard et al. (2015a). About a week prior to testing, participants undertook a complete preliminary session where they performed short (<5 s) treadmill sprints at increasing intensities wearing a facemask for habituation (i.e., with the hypoxic system turned off), with full recovery and until being comfortable with the running technique required (which generally necessitated 7–10 trials). Then they performed three maximal 5-s single sprints, separated by 2 min of passive recovery, and after 5 min of rest, the repeated-sprint exercise test in full. All of them satisfied the criteria of having a coefficient of variation < 2.2% for distance covered across three successive trials (Girard et al., 2015b). Strong verbal encouragement was given during all maximal efforts. Participants were also thoroughly familiarized with the neuromuscular function assessment protocol (see *Neuromuscular Function*) until they felt accustomed with the equipment (i.e., coefficient of variation in three successive KE trials for peak RFD and maximal torque with “fast” and “hard” instructions lower than 5 and 3%, respectively).

Participants reported to the laboratory (well-ventilated at a constant temperature of ~25°C and 40% relative humidity) on three different occasions (~1 h; counterbalanced randomized crossover design in double-blind fashion) at least 3–4 days apart to complete an experimental session. This involved performing a repeated-sprint running protocol on a sprint treadmill (ADAL3D-WR, Medical Development – HEF Tecmachine, Andrézieux-Bouthéon, France), allowing participants to produce realistic acceleration and high running velocities (Morin et al., 2010). Participants performed their trials at the same time of the day (± 1 h) and wore similar sports gear (running shoes, short, and T-shirt). They were instructed to maintain their normal diet (i.e., avoiding any nutritional supplements or alcohol consumption), sleeping (i.e., ≥7 h/night) and training (i.e., avoiding vigorous exercise 24 h before every trial) habits during the 1–2 weeks period of testing to prevent any possible interference on their sprinting abilities. Participants were instructed to drink 4–6 mL of water per kilogram of body mass every 2.5 h on the day before each experimental session to ensure euhydration at the start of exercise. They were permitted to drink *ad libitum* during the warm-up procedure.

### Experimental protocol

Upon arrival on testing days, participants were instrumented and pre-exercise (Pre-tests) neuromuscular function assessment (see *Neuromuscular Function*) was conducted in normoxia. Thereafter, they completed a running warm-up (i.e., on the sprint treadmill with participants breathing ambient air) consisting of 5 min of running at 10 km.h^−1^, followed by 10 min of sprint-specific muscular warm-up exercises [i.e., 3 × (skipping, high knee, butt-kick, high heels for ~10 s with 30-s walking in between), followed by 3 × (3 steps accelerations at a subjective “sense of effort” of 7, 8, and 9 on a modified Borg 10 scale), then by 2 × (3-s sprints at a subjective “sense of effort” of 8 and 9)] (Christian et al., 2014). Afterwards, three maximal 5-s single sprints, separated by 2 min of passive recovery, were completed. After a facemask connected to a portable hypoxic generator (Altitrainer, SMTEC SA, Nyon, Switzerland) had been attached on participants, they were allowed 5-min of free cool-down prior to the repeated-sprint protocol. This exercise consisted of performing eight, 5-s “all-out” sprints interspersed with 25 s of passive rest and was randomly conducted in normoxia (NM; FiO_2_ = 20.9%), in low and high simulated altitudes (normobaric hypoxia) of ~1800 m (LA; FiO_2_ = 16.8%) and ~3600 m (HA; FiO_2_ = 13.3%), respectively. Normobaric hypoxia was obtained by mixing nitrogen into ambient air under control of FiO_2_. During recovery periods, participants stood quietly on the treadmill. Repeated-sprint ability was assessed from covered distance data using three scores: the largest (i.e., during the first sprint in all cases) distance ran, the cumulated distance covered over the eight sprints (i.e., sum of the eight sprints) and the sprint decrement score [i.e., ([cumulated distance/(largest distance × 8)]–1) × 100] (Girard et al., 2011). Finally, the neuromuscular function assessment was repeated (Post-tests) in normoxia (i.e., participants took off the facemask 25 s after completion of the last sprint) and was started exactly 1 min after the repeated-sprint exercise protocol ended.

### Responses to exercise

Heart rate and SpO_2_ were monitored and estimated, respectively, via a Polar transmitter-receiver (Wearlink T-31, Polar Electro Oy, Kempele, Finland) and non-invasive pulse oximetry using a finger probe (Palmsat, 2500, NONIN Medical Inc., Plymouth, MI, USA). Together with heart rate and SpO_2_, ratings of perceived exertion were recorded using the Borg 6–20 scale (i.e., 6 = no exertion at all, 20 = maximal exertion) exactly 10 s following each sprint (i.e., peak values likely to be obtained). Additionally, SpO_2_ was recorded between before the warm-up and 4 min after the last sprint. These time points corresponded to the end of pre- and post-tests.

### Neuromuscular function

Neuromuscular test sessions began by the completion of three successful MVCs, all brief (~5 s) and separated by ≥30 s of rest, with a twitch delivered over the isometric plateau. Participants were instructed to increase torque production over a 1-s period, hold it for 3–4 s and then relax before completing the next contraction. Thereafter, participants were instructed to perform “explosive” MVCs (separated by ≥20 s). During all brief MVCs trials the participants were carefully instructed to contract “as fast as possible” for ~1 s from a fully relaxed state, in an attempt to achieve at least 90% of their MVC torque. Participants were asked to avoid any countermovement before torque onset; i.e., they were reminded not to flex the knee immediately prior to KE. They were strongly encouraged with verbal feedback and a visual display of the torque production. Contractions that had any discernable countermovement or pre-tension (i.e., change of baseline torque of >1.5 Nm during the 100 ms before contraction onset; Girard et al., 2014) were discarded and another attempt was made. To provide biofeedback on whether a countermovement had occurred, the resting torque level was displayed on a sensitive scale. The slope of the torque–time curve (10 ms time constant) was displayed throughout testing and the peak slope was used to provide visual performance feedback to participants after each contraction. Pre-tests assessment was preceded by a warm-up consisting of 10 isometric contractions of ~3–5 s in duration interspaced with ~10–20 s of recovery. Contraction intensity was progressively self-adjusted by the participant to attain maximal torque in the last three contractions.

### Recordings

#### Torque measurements

KE torque was measured with participants seated upright on a custom-built adjustable chair with the hips and knees flexed at 90°. Restraining straps placed across the chest and hips secured the participants in the chair to prevent extraneous movement, while the dynamometer (Captels, St Mathieu de Treviers, France) was attached 3–5 cm above the tip of the lateral malleoli. During all contractions the torque signals were amplified, sent through an A/D board and sampled at 2000 Hz by commercially available hardware and software (MP35 and BSL Pro Version 3.6.7, Biopac Systems Inc., Santa Barbara, USA).

#### Electromyography

The EMG activity of the VL and *rectus femoris* (RF) muscles was recorded via bipolar Ag/AgCl electrodes (Ambu Blue sensor T, Ambu A/S, Denmark; diameter = 9 mm; inter-distance electrode = 30 mm) fixed longitudinally over the muscle bellies. The reference electrode was attached to the right wrist. Low impedance between the two electrodes was obtained by abrading the skin with emery paper and cleaning with alcohol. The position of the electrodes was marked for consistent placement. EMG signals were amplified (gain = 1000), filtered (band-width frequency 30–500 Hz) and recorded (sampling frequency = 2000 Hz) by commercially available hardware (Biopac MP35, systems Inc., Santa Barbara, CA) and software (Acqknowledge 3.6.7, Biopac Systems Inc., Santa Barbara, CA).

#### Motor nerve stimulation

Femoral nerve stimulations (400 V, rectangular pulse of 0.2 ms) were delivered by a high-voltage stimulator (Digitimer DS7AH; Digitimer, Hertfordshire, UK) via a cathode electrode (diameter of 5 mm) placed in the inguinal crease and an anode (5 × 10 cm; Medicompex, SA, Ecublens, Switzerland) in the gluteal fold. The intensity of stimulation was determined during the familiarization test session using a passive isometric recruitment curve (Racinais et al., 2013). Briefly, the stimulation intensity was increased by 10-mA increments until a maximal peak twitch torque was achieved and then a further increased by 50% to ensure constant supramaximal stimulation throughout the protocol.

### Data analysis

All analyses were performed using Spike 2 Software (Cambridge Electronic Design, Cambridge, UK). The MVC torque was defined as the maximum value recorded for 1 s when the torque had reached a plateau (before the superimposed twitch), and the RMS of the EMG activity was computed during the same 1-s period (RMS_MAX_). Similarly, the peak-to-peak amplitude of superimposed maximum compound action potential (M-wave) responses was measured for each agonist muscle, and RMS_MAX_ was divided by M-wave to give a ratio RMS_MAX_/M-wave.

The contractile RTD (expressed as Nm.s^−1^) was derived from the “explosive” MVC measurements, as the average slope of the initial time phase of the torque-time curve at 0–30, 0–50, 0–100, and 0–200 ms, relative to the onset of contraction (Aagaard et al., 2002; Suetta et al., 2004; Thorlund et al., 2009) using a custom written program (Spike 2 Software, Cambridge Electronic Design, Cambridge, UK). The onset of muscle contraction was defined as the time point at which the torque curve exceeded baseline by >4.5 Nm, corresponding to ~2.5% of MVC torque values (Andersen et al., 2010). The peak RTD was defined as the peak Δtorque/Δtime achieved during the initial 200 ms of the isometric contraction (de Oliveira et al., 2013; Girard et al., 2014). In addition, the rate of muscle activation (expressed as mV.s^−1^) was measured as the raw RMS activity increase obtained at similar time intervals relative to onset integration (i.e., activity). The onset of EMG integration was shifted 50 ms before the onset of contraction to account for the presence of electromechanical delay (Aagaard et al., 2002; Girard et al., 2013). The RTD and EMG rise were also normalized relative to maximal MVC torque (%MVC) and maximal EMG activity (%RMS_MAX_/M-wave). The mean over three trials was used for further analysis for each parameter.

### Statistical analysis

Values are expressed as means ± SD. Two-way repeated-measures ANOVAs [Time (Pre-tests vs. Post-tests) × Condition (NM, LA vs. HA)] were used to compare torque and muscle activation data for each time window (0–30, 0–50, 0–100, and 0–200 ms) independently for absolute and relative changes. Outcome variables were tested using Mauchly's procedure for sphericity. Whenever the data violated the assumption of sphericity, *P*-values and adjusted degrees of freedom based on Greenhouse-Geisser correction were reported instead. Where significant effects were established, pairwise differences were identified using the Bonferroni *post-hoc* analysis procedure adjusted for multiple comparisons. For each ANOVA, partial eta-squared was calculated as measures of effect size. Values of 0.01, 0.06, and above 0.14 were considered as small, medium, and large, respectively. All statistical calculations were performed using SPSS statistical software V.21.0 (IBM Corp., Armonk, NY, USA). The significance level was set at *P* < 0.05.

## Results

### Repeated-sprint ability and responses to exercise

**Figure 1:**
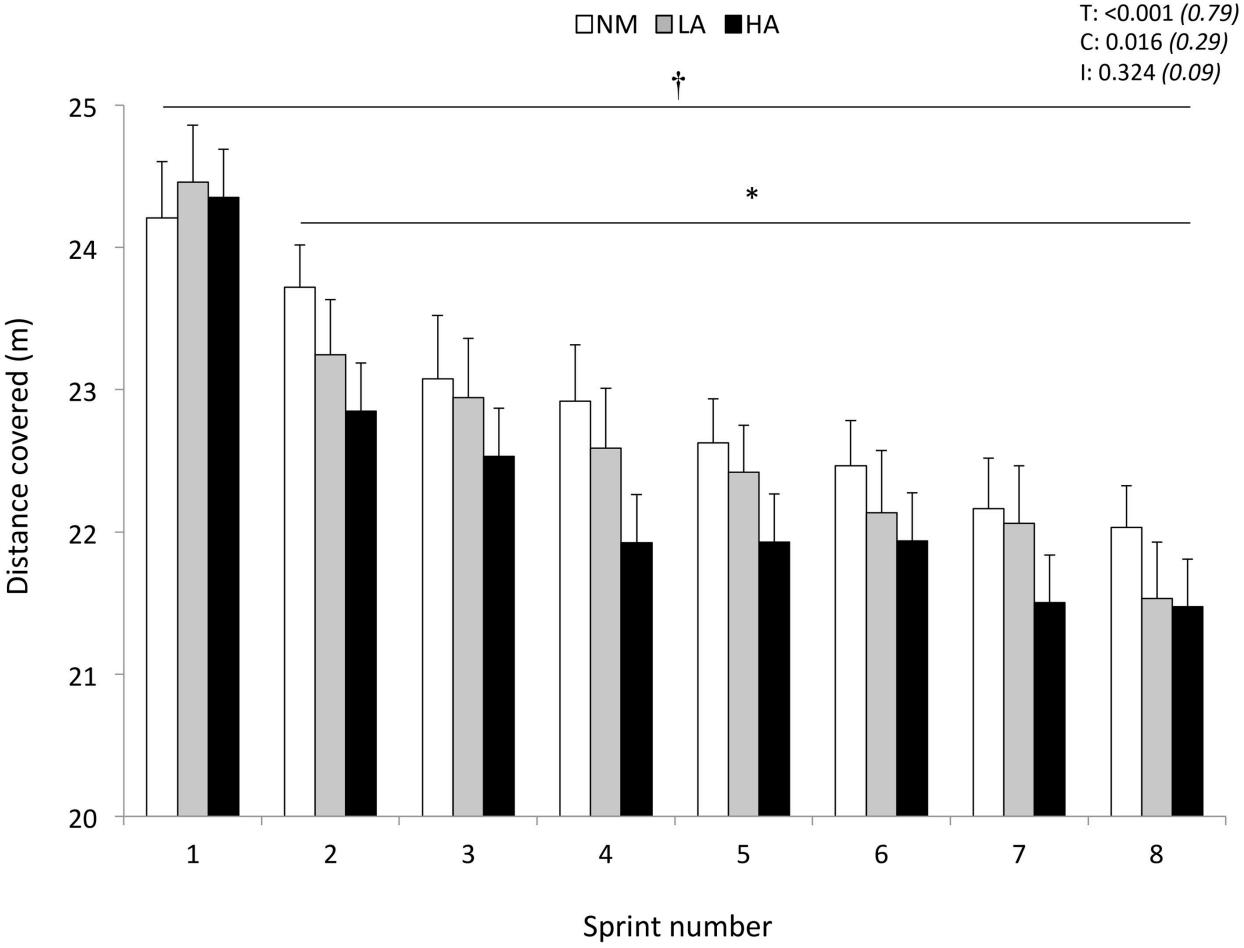
**Distance covered during the repeated-sprint ability test**. Data are presented in normoxia (NM; FiO_2_ = 20.9%), at low (LA; FiO_2_ = 16.8%), and high (HA; FiO_2_ = 13.3%) normobaric hypoxia. Values are mean ± SD (*n* = 13). T, C, and I respectively refer to ANOVA main effects of time, condition, and interaction between these two factors with *P*-value and partial eta-squared in parentheses. ^*^Significantly different from sprint 1 (all conditions pooled), *P* < 0.05. ^†^NM different from HA (all sprints pooled), *P* < 0.05.

Distance covered during the first 5-s sprint was similar (24.2 ± 1.4, 24.5 ± 1.5, and 24.4 ± 1.7 m for NM, LA, and HA, respectively; *P* > 0.05) across conditions (Figure 1). In reference to sprint 1, distance covered decreased from sprint 2 onwards (*P* < 0.001), independently of the condition (*P* = 0.324). The averaged values of distance covered for sprints 1–8 were lower in HA (22.3 ± 1.3 m) compared to NM (22.9 ± 1.2 m; *P* = 0.044) but not LA (22.7 ± 1.3 m; *P* = 0.183), with also no difference between NM and LA (*P* = 0.710). A larger sprint decrement score occurred in HA (−7.8±3.6%) vs. NM (−5.3±1.9%; *P* = 0.015) but not LA (−6.3±3.5%; *P* = 0.060), with also no difference between NM and LA (*P* = 0.237). Compared to NM (183.2 ± 9.3 m), the cumulated distance covered over the eight sprints was shorter in HA (178.5 ± 10.7 m; *P* = 0.014) but not in LA (181.4 ± 10.3 m; *P* = 0.056), with also no difference between NM and LA (*P* = 0.240).

Whereas, it did not change in NM (96.8 ± 0.8 vs. 96.0 ± 1.6%; *P* = 0.455), SpO_2_ values decreased from the first to the last sprint in LA (95.0 ± 2.0 vs. 89.7 ± 4.0%; *P* = 0.050) and HA (88.8 ± 2.5 vs. 81.8 ± 4.5%; *P* = 0.001) (Table 1). Compared to pre-tests (96.9 ± 0.4%), SpO_2_ values were not different among conditions during post-tests after completion of the repeated-sprint ability protocol (96.2 ± 0.5%; all conditions pooled, *P* > 0.05). Heart rate (NM: 150 ± 15 vs. 176 ± 12 bpm; LA: 154 ± 9 vs. 176 ± 9 bpm; HA: 154 ± 12 vs. 174 ± 13 bpm, all *P* < 0.001) and ratings of perceived exertion (NM: 11.8 ± 1.4 vs. 18.5 ± 1.2 points; LA: 11.3 ± 1.4 vs. 18.1 ± 1.6 points; HA: 11.5 ± 1.8 vs. 19.1 ± 0.8 points, all *P* < 0.001) increased from sprint 1 to sprint 8, irrespective of the environmental condition (*P* = 0.256). Higher ratings of perceived exertion values were recorded for the average of eight sprints in HA (15.8 ± 0.3 points) vs. LA (15.2 ± 0.3 points; *P* = 0.006), but not NM (15.5 ± 0.3 points; *P* = 0.452).

**Table 1:** **Changes in responses to exercise during the repeated-sprint ability test in normoxia (NM; FiO_2_ = 20.9%), under low (LA; FiO_2_ = 16.8%) and high (HA; FiO_2_ = 13.3%) normobaric hypoxia**.

**Variables**	**Sprint number**								**ANOVA *(Partial eta-squared)***
	**1**	**2**	**3**	**4**	**5**	**6**	**7**	**8**	
**SpO_2_ (%)**									
NM	96.8±0.8	96.4±1.9	96.3±1.9	95.8±2.0	96.2±2.1	95.8±2.0	95.8±1.5	96.0±1.6	T < 0.001 *(0.60)*
LA^#^	95.0±2.0	94.3±3.2	92.4±3.4^*^	91.3±2.9^*^	91.5±3.0^*^	91.5±3.0	89.6±4.4^*^	89.7±4.0^*^	C < 0.001 *(0.85)*
HA^#, †^	88.8±2.5	86.1±4.5	84.5±4.1^*^	84.0±4.9^*^	83.6±5.2^*^	83.2±4.6^*^	82.5±5.3^*^	81.8±4.5^*^	I = 0.016 *(0.24)*
**HR (bpm)**									
NM	150±15	164±13^*^	169±13^*^	171±12^*^	174±12^*^	175±11^*^	175±11^*^	176±12^*^	T < 0.001 *(0.92)*
LA	154±9	166±11^*^	172±9^*^	175±10^*^	176±10^*^	176±11^*^	176±10^*^	176±9^*^	C = 0.427 *(0.07)*
HA	154±12	166±15^*^	171±13^*^	174±14^*^	174±13^*^	175±12^*^	174±12^*^	174±13^*^	I = 0.187 *(0.12)*
**RPE (POINTS)**									
NM	11.8±1.4	13.0±1.1^*^	14.3±1.4^*^	15.5±1.5^*^	16.4±1.7^*^	17.1±1.6^*^	17.7±1.5^*^	18.5±1.2^*^	T < 0.001 *(0.95)*
LA	11.3±1.4	12.7±1.1^*^	13.8±1.2^*^	15.1±1.1^*^	16.1±1.2^*^	17.0±1.5^*^	17.6±1.4^*^	18.1±1.6^*^	C = 0.034 *(0.25)*
HA^†^	11.5±1.8	13.0±1.6^*^	14.4±1.6^*^	15.7±1.6^*^	16.7±1.4^*^	17.5±1.3^*^	18.4±1.1^*^	19.1±0.8^*^	I = 0.256 *(0.10)*

*Mean ± SD (n = 13)*.

*SpO_2_, arterial O_2_ saturation; HR, heart rate; RPE, ratings of perceived exertion*.

* *Significantly different from sprint 1 (P < 0.05)*.

*^#^and significantly different from NM and LA, respectively (P < 0.05)*.

### Maximal strength

Compared to NM (−9 ± 7%), a larger (*P* < 0.05) reduction in MVC torque occurred from pre- to post-exercise in HA (−14 ± 9%; *P* = 0.021) but not in LA (−12 ± 7%; *P* = 0.270), with also no differences between LA and HA (*P* = 0.340) (Table 2). During MVCs, raw EMG signals of both VL and RF muscles were lower at post- relative to pre-, with no difference between conditions. Peak-to-peak M-wave amplitudes for both VL and RF muscles did not change. A global reduction of the RMS_MAX_/M-wave ratio occurred from pre- to post-exercise for the RF (−14 ± 11%; *P* = 0.002), while failing to reach statistical significance for the VL (−8 ± 11%; *P* = 0.075).

**Table 2:** **Neuromuscular parameters recorded during brief explosive maximal knee extension before (Pre-tests) and after (Post-tests) repeated sprinting in normoxia (NM; FiO_2_ = 20.9%), under low (LA; FiO_2_ = 16.8%) and high (HA; FiO_2_ = 13.3%) normobaric hypoxia**.

**Variables**	**Pre-tests**			**Post-tests**			**ANOVA ***(Partial eta-squared)*****		
	**NM**	**LA**	**HA**	**NM**	**LA**	**HA**	**Time**	**Condition**	**Interaction**
MVC torque (Nm)	271.0±45.6	269.5±41.9	270.1±42.3	246.5±35.4^*^	237.6±39.0^*^	232.3±40.8^*^^#^	<0.001 *(0.77)*	0.085 *(0.19)*	0.021 *(0.28)*
Peak RTD (Nm.s)	1018±122	1074±208	986±195	965±133^*^	948±152^*^	942±199^*^	0.031 *(0.33)*	0.024 *(0.11)*	0.040 *(0.24)*
RMS_MAX__VL (mV)	0.604±0.112	0.599±0.110	0.597±0.111	0.568±0.107	0.524±0.093	0.553±0.110	<0.001 *(0.59)*	0.159 *(0.14)*	0.117 *(0.16)*
RMS_MAX__RF (mV)	0.449±0.173	0.451±0.158	0.449±0.160	0.403±0.162	0.398±0.147	0.395±0.169	<0.001 *(0.65)*	0.927 *(0.06)*	0.777 *(0.13)*
M-wave_VL (mV)	11.4±4.5	12.0±4.5	11.4±4.6	11.5±4.5	11.3±4.4	11.9±4.9	0.903 *(0.01)*	0.905 *(0.08)*	0.176 *(0.15)*
M-wave_RF (mV)	12.4±2.0	12.6±3.2	12.4±2.0	13.0±2.1	12.5±1.4	12.8±1.7	0.436 *(0.051)*	0.914 *(0.07)*	0.570 *(0.05)*
RMS_MAX_/M-wave_VL (mV)	0.054±0.027	0.052±0.025	0.056±0.030	0.051±0.025	0.049±0.029	0.048±0.022	0.075 *(0.24)*	0.643 *(0.04)*	0.395 *(0.07)*
RMS_MAX_/M-wave_RF (mV)	0.038±0.018	0.039±0.020	0.037±0.017	0.033±0.016	0.033±0.013	0.031±0.015	0.002 *(0.57)*	0.053 *(0.05)*	0.813 *(0.02)*

*Mean ± SD (n = 13)*.

*MVC torque, maximal voluntary contraction torque; Peak RTD, peak rate of torque development; RMS_MAX_, M-wave and RMS_MAX_/M-wave represent the average of root mean square, maximal M-waves, and normalized electromyogram activity of vastus lateralis (VL) and rectus femoris (RF) muscles*.

* *Significantly different from Pre-tests (P < 0.05)*.

# *Significantly different from NM (P < 0.05)*.

### Rapid muscle characteristics

**Figure 2:**
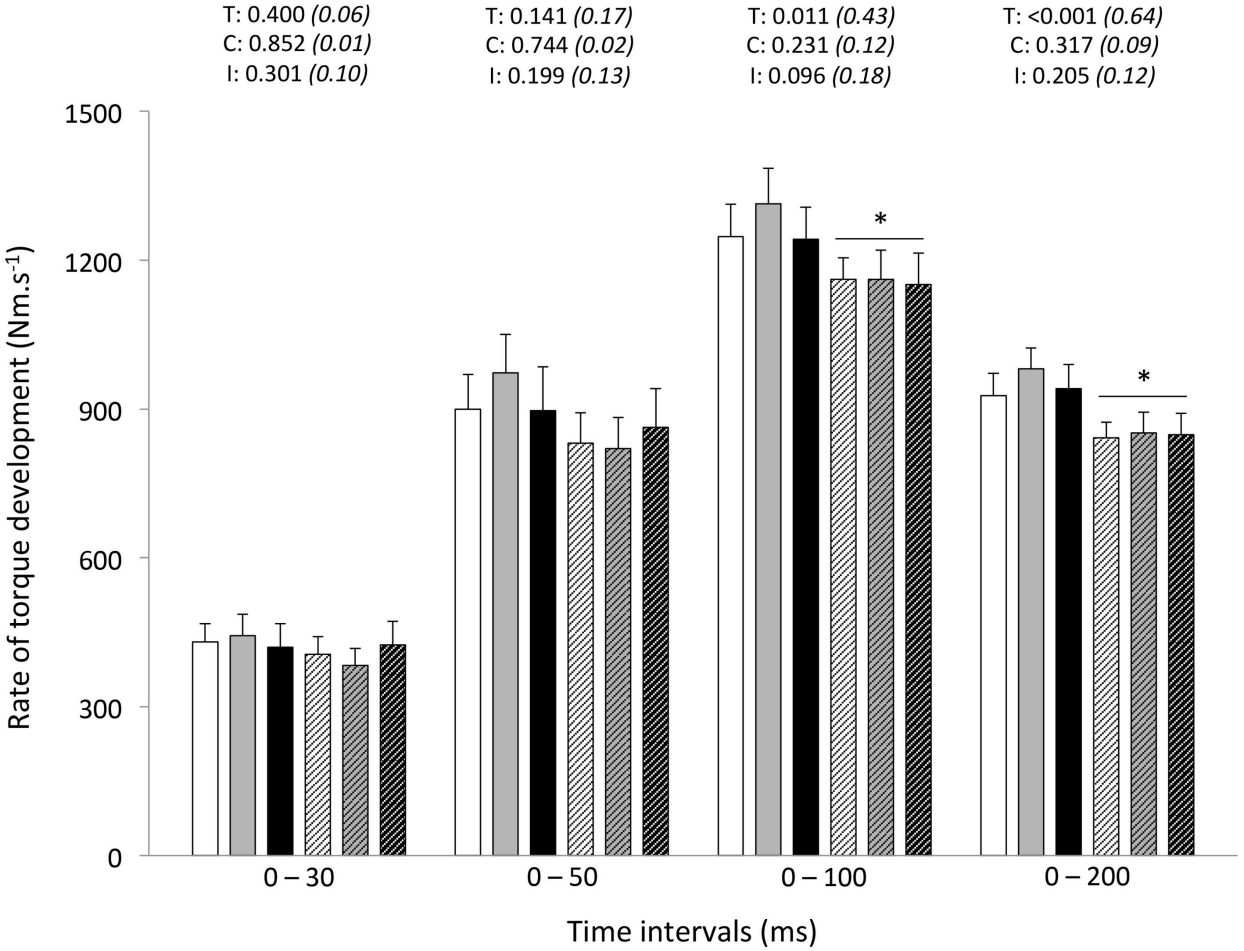
**Rate of torque development (absolute values) during explosive isometric knee extension obtained at 0–30, −50, −100, and −200 ms prior to (Pre-tests; full bars) and following (Post-tests; dashed bars) repeated sprinting in normoxia (NM; FiO_2_ = 20.9%; white bars), at low (LA; FiO_2_ = 16.8%; gray bars) and high (HA; FiO_2_ = 13.3%; black bars) normobaric hypoxia**. Values are mean ± SD (*n* = 13). T, C, and I respectively refer to ANOVA main effects of time, condition, and interaction between these two factors with *P*-value and partial eta-squared in parentheses. ^*^Significantly different from Pre-tests, *P* < 0.05.

**Figure 3:**
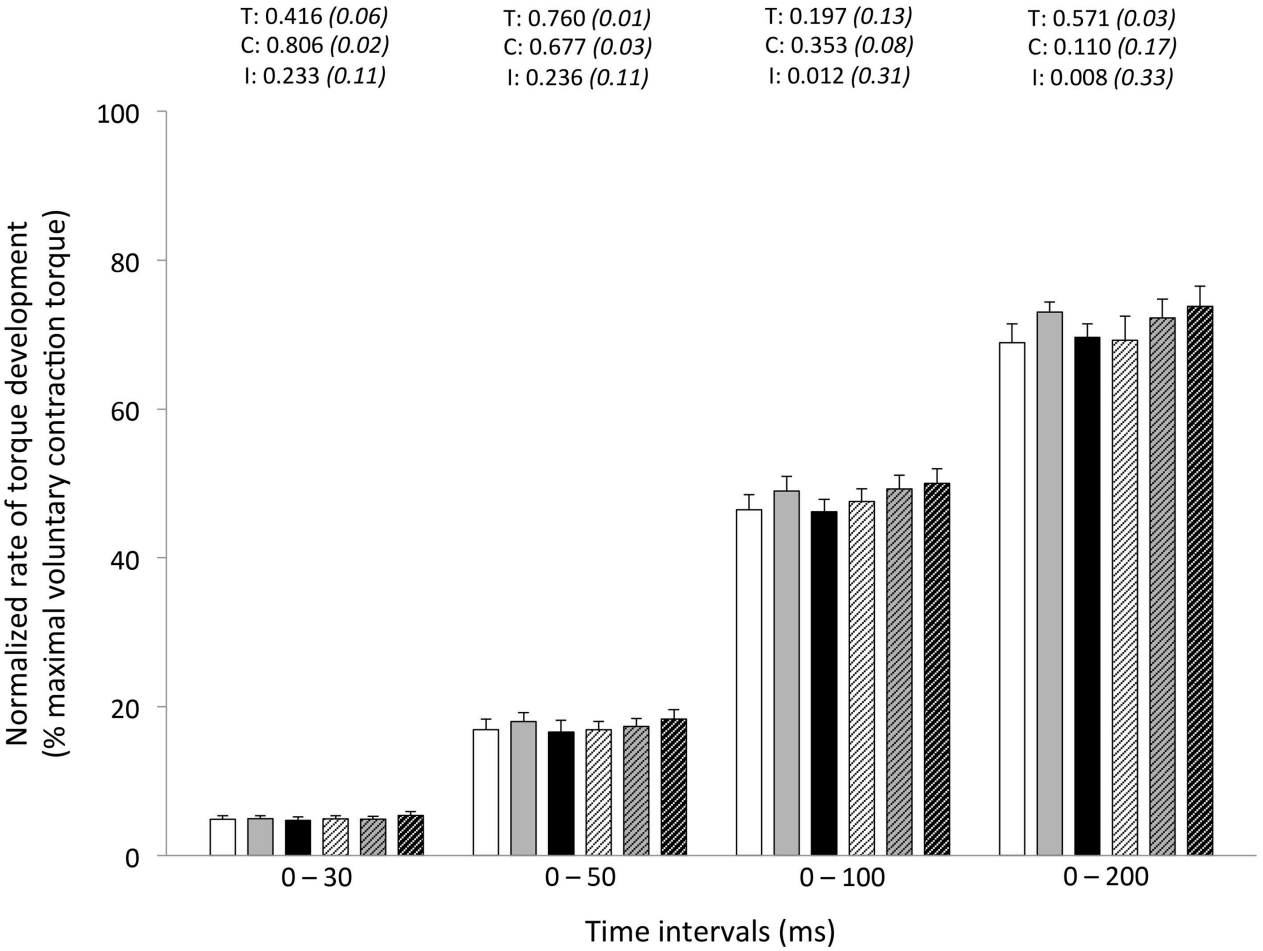
**Normalized rate of torque development (% maximal voluntary contraction torque) during explosive isometric knee extension obtained at 0–30, −50, −100, and −200 ms prior to (Pre-tests; full bars) and following (Post-tests; dashed bars) repeated sprinting in normoxia (NM; FiO_2_ = 20.9%; white bars), at low (LA; FiO_2_ = 16.8%; gray bars) and high (HA; FiO_2_ = 13.3%; black bars) normobaric hypoxia**. Values are mean ± SD (*n* = 13). T, C, and I respectively refer to ANOVA main effects of time, condition and interaction between these two factors with *P*-value and partial eta-squared in parentheses.

**Figure 4:**
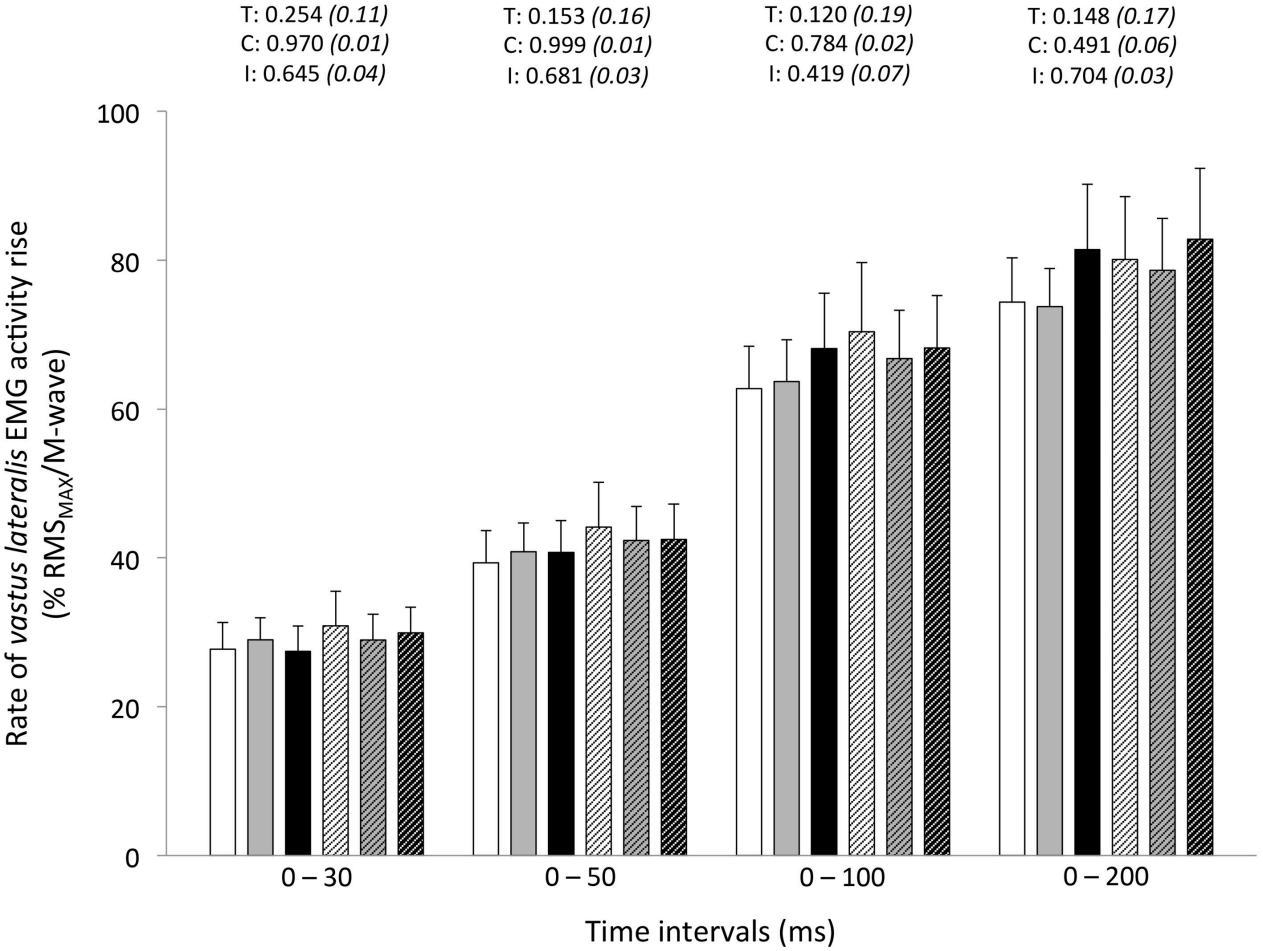
**Rate of *vastus lateralis* EMG activity rise (% RMS_MAX_/M-wave) during explosive isometric knee extension obtained at 0–30, −50, −100, and −200 ms prior to (Pre-tests; full bars) and following (Post-tests; dashed bars) repeated sprinting in normoxia (NM; FiO_2_ = 20.9%; white bars), at low (LA; FiO_2_ = 16.8%; gray bars) and high (HA; FiO_2_ = 13.3%; black bars) normobaric hypoxia**. Values are mean ± SD (*n* = 13). T, C, and I respectively refer to ANOVA main effects of time, condition and interaction between these two factors with *P*-value and partial eta-squared in parentheses.

**Figure 5:**
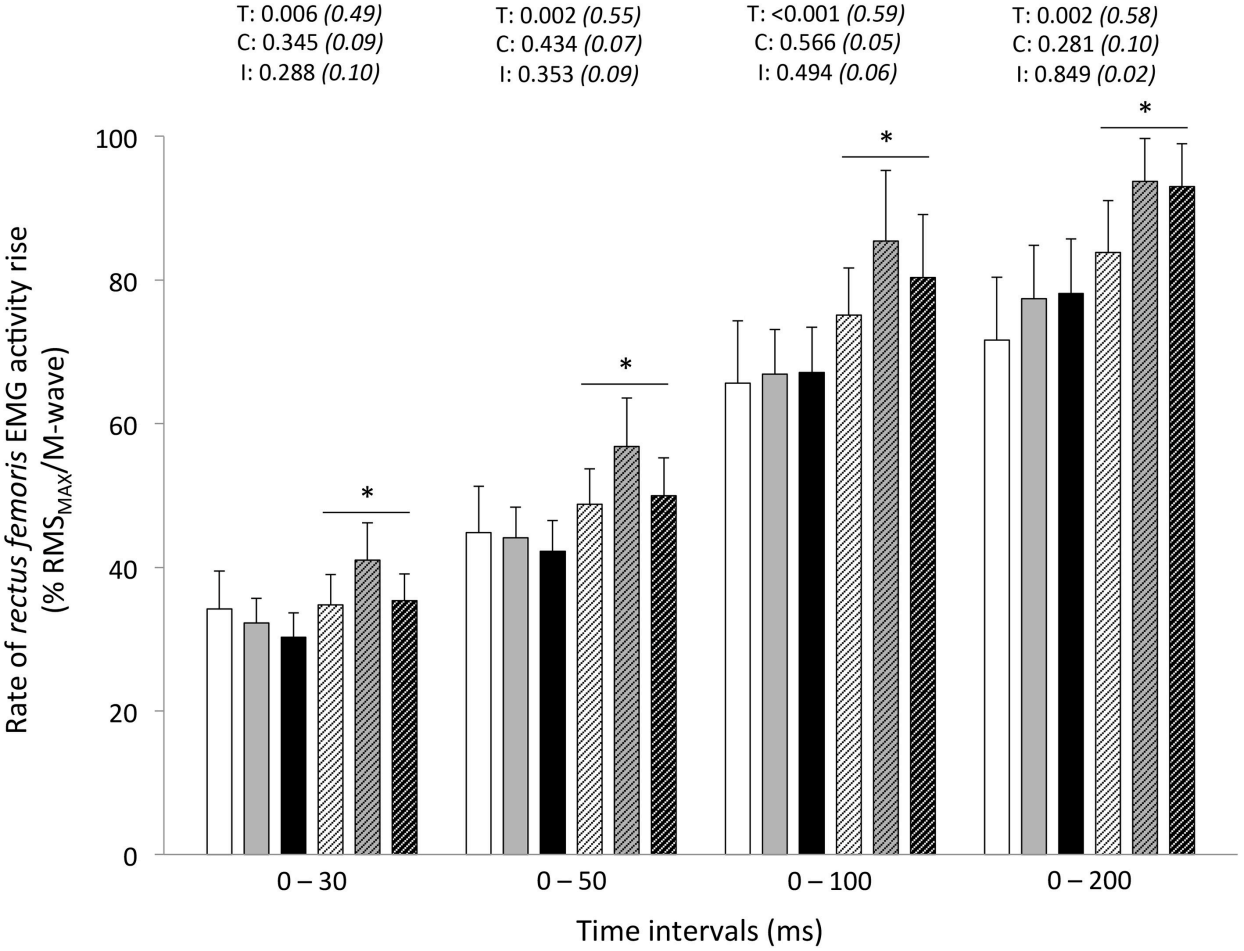
**Rate of *rectus femoris* EMG activity rise (% RMS_MAX_/M-wave) during explosive isometric knee extension obtained at 0–30, −50, −100, and −200 ms prior to (Pre-tests; full bars) and following (Post-tests; dashed bars) repeated sprinting in normoxia (NM; FiO_2_ = 20.9%; white bars), at low (LA; FiO_2_ = 16.8%; gray bars) and high (HA; FiO_2_ = 13.3%; black bars) normobaric hypoxia**. Values are mean ± SD (*n* = 13). T, C, and I respectively refer to ANOVA main effects of time, condition and interaction between these two factors with *P*-value and partial eta-squared in parentheses. ^*^Significantly different from Pre-tests, *P* < 0.05.

Peak RTD (all conditions pooled: −6 ± 11%; *P* = 0.031) was significantly reduced from pre- to post-exercise (Table 2). Reduction in RTD (absolute values) occurred within the 0–100 (−8 ± 9%; *P* = 0.011) and 0–200 ms (−10 ± 8%; *P* < 0.001) epochs after contraction onset, independent of the condition (*P* > 0.23; Figure 2). No differences in RTD (relative values) were observed after normalization to MVC torque (*P* > 0.197; Figure 3). Furthermore, the relative rates of EMG rise for VL muscle for any epochs throughout the experimental protocol were not different between conditions (*P* > 0.49) and there were no interaction (*P* > 0.42) or time effects (*P* > 0.12) (Figure 4). The rate of EMG rise for RF muscle increased during the periods 0–30 (+22 ± 26%; *P* = 0.006), 0–50 (+25 ± 28%; *P* = 0.002), 0–100 (+27 ± 27%; *P* < 0.001), and 0–200 ms (+23 ± 2%; *P* = 0.002) post-exercise, independently of the condition (*P* > 0.23; Figure 5).

## Discussion

### Repeated-sprint performance and responses to exercise

Distance ran during the first 5-s sprint was similar in all conditions, as an enhanced anaerobic energy release can compensate for the reduced aerobic ATP production during short maximal efforts in hypoxic conditions (Calbet et al., 2003). Compared to NM, a larger sprint decrement score and a shorter cumulated distance covered from sprint 1 to 8 occurred in HA, but not in LA, with also no difference between NM and LA. Consistent with previous repeated sprinting literature (Bowtell et al., 2014), our data therefore show that performance fatigability was significantly exacerbated relative to NM only under our severer hypoxic condition. The question of whether the same is true when completing identical repeated sprinting protocol at natural altitude (i.e., hypobaric hypoxia), known to induce severer physiological responses (Millet et al., 2012) and eventually larger neuromuscular alterations, has not been specifically addressed. Hence, the decrease in air density upon ascent to terrestrial altitude reduces air resistance, which is likely to decrease the energy cost of running at high velocities, and thereby improve single sprint performance (Levine et al., 2008). When sprints are repeated, however, hypobaric hypoxia would induce higher work of breathing responses and more detrimental neuromuscular consequences than exposure to gas mixtures lowering FiO_2_. Despite lower SpO_2_ values in the severer hypoxic condition, it is interesting to observe in this study that sensations that regulated the integrity of the performer (ratings of perceived exertion or perceived fatigability) and associated heart rate responses did not differ between SL and HA.

### Neuromuscular parameters during maximal contractions

Along with lower distance covered during the repeated-sprint test, post-exercise reduction in maximal KE torque, as measured from brief MVCs, was ~5% larger in HA (−14%) compared to NM (−9%). This later result is in line with a previous study where the decrease in KE MVC torque under hypoxia (FiO_2_ = 14%) was 6% larger in reference to normoxia after the completion of 15 5-s cycling sprints interspersed with 25-s of rest (Billaut et al., 2013). Despite not supported by statistical analysis, our novel finding was that a graded effect of hypoxia was visible for strength losses post-repeated sprinting with progressively larger values (−9, −12, and −14%) with increasing severities of acute hypoxia. This extends similar findings following maximal intermittent dynamic leg extension where KE MVC torque losses increased (yet non-significantly) with hypoxia severity (−18, −19, and −27% in FiO_2_ = 21, 14, and 10%, respectively) (Christian et al., 2014). Contrastingly, performing sets of intermittent, isometric, quadriceps contractions at 60% of MVC force to task failure in normoxia, mild hypoxia, moderate hypoxia and severe hypoxia (FiO_2_ = 21, 16, 13, and 10%) resulted in ~30% declines in MVC force in all conditions, despite large differences in time-to-task failure (24.7 vs. 15.9 min in FiO_2_ = 21 vs. 10%) (Goodall et al., 2010). Potentially, differences in exercise mode/duration/intensity and individuals' tolerance to hypoxic stress, in turn affecting the magnitude of reduction in convective O_2_ transport, may explain aforementioned diverging results on MVC torque and further confirm that fatigue is task-dependent.

Also worth noticing, the effect of fatigue on M-wave changes during repeated sprinting is not clear-cut in literature, with reports of decreased (Perrey et al., 2010), unchanged (Girard et al., 2013), and increased (Racinais et al., 2007) amplitudes. Consequently, normalizing the raw EMG amplitude to a maximum compound action potential (M-wave) is a methodological requirement allowing control for any changes in neuromuscular junction and sarcolemma excitation, and hence, enhancing the sensitivity of EMG amplitude measurements. In this study, maximal normalized EMG activity (RMS_MAX_/M-wave ratio) was dampened during MVCs in VL (−8%; albeit not significantly different) and RF (−14%) muscles. This suggests that the magnitude of the efferent motor outflow reaching the KE was adversely affected by the repetition of eight maximal sprints, confirming previous observations (Girard et al., 2015a; Brocherie et al., 2015b), while severity of hypoxic exposure had only minimal effect.

### Rapid muscle force characteristics

In addition to the post-exercise decrement in MVC torque, this study is unique as far as we are aware in reporting that the altitude severity (at least to 3600 m) did not modify the post-exercise RTD responses: the early phase RTD (30 and 50 ms) did not change, whereas the decline in peak RTD and absolute RTD values in the late phase (100 and 200 ms) of muscle contraction was similar between the normoxic and hypoxic conditions. In hot and cool conditions, a global (i.e., at all time intervals) downward-shift in the contractile RTD occurred after repeated cycling sprints (Girard et al., 2013). As it was unrelated to environmental conditions (i.e., modest hyperthermia), this was taken to reflect an overall fatigue-induced reduction in rapid muscle force characteristics. In the present study adopting a running mode, the difference between early (unchanged) and late (decreased) phases absolute RTDs may relate to the relative influence of passive stiffness in serial/lateral force transmission structures, myofiber cross-bridge kinetics and neural drive (Edman and Josephson, 2007). With late phase RTD more likely be affected by the fiber type composition (Penailillo et al., 2014), a more pronounced fatigue of faster fiber types after repeated running sprinting (Girard et al., 2015a), in turn associated with slower cross-bridge kinetics (Hamada et al., 2003), may dictate observed magnitude of post-exercise alterations in absolute RTD. Importantly, the exercise-related decline seen for the late-phase absolute RTD values occurred regardless of the environmental condition. In line with these findings, we have recently reported that heat stress does not exacerbate alterations in rapid muscle torque production capacity of KE neither after repeated cycling sprints (Girard et al., 2013) nor prolonged tennis playing (Girard et al., 2014).

In this study, when controlling for fatigue-induced reduction in maximal strength, we failed to demonstrate significant reductions in explosive strength post-exercise at any time interval. In fact, RTD is increasingly related to maximal muscle force, and reliance on muscle contractile properties decreases, as the time (mostly from 90 ms) from the onset of contraction increases (Andersen and Aagaard, 2006). The time interval in which RTD is determined influences the nature of the association between maximal muscle force and RTD and, in turn, the nature of fatigue-induced responses. By using different experimental designs (i.e., resistance training and verbal instructions during protocol), however, others have questioned the direct relationship between maximal force and RTD (Griffin and Cafarelli, 2005; Holtermann et al., 2007). Regardless, our data show the influence of maximal strength in maintaining normalized RFD values at all time intervals after repeated sprinting in differing hypoxic conditions.

### Muscle activation rise

Whereas maximal VL muscle activation capacity (RMS_MAX_/M-wave ratio) was impaired by sprints repetition, the ability to rapidly activate the VL muscle, as inferred from the rate of EMG amplitude rise, did not change in our study. Consistently, whole-body exercise studies, for instance following simulated team- (football: Thorlund et al., 2009; handball: Thorlund et al., 2008) and racket- (tennis: Girard et al., 2014) sport matches or a repeated cycling sprint protocol (Girard et al., 2013), show that reductions in RTDs are generally not associated with significant decreases in EMG values of quadriceps muscles. Opposite results have been reported by Morel et al. (2015) after the completion of 20 sets of 6-s isokinetic maximal KE at 240° s^−1^, starting every 30 s. In their study, reductions of RTD were associated with a dampened activation capacity of the VL muscle in the early phase of muscle contraction, whereas participants were capable of producing similar maximal activation levels (Morel et al., 2015). Methodological differences (e.g., various time intervals, manual vs. automated methods to detect contraction onset; instructions to the participants during the contraction execution) preclude meaningful comparisons of EMG responses between studies.

In our study, the fact that RF muscle activation rise increased (at all time intervals) from pre- to post-exercise, despite significant alteration of the ability to maximally activate this muscle, is an interesting observation. Considering the different activation sequences of this bi-articular muscle during sprinting (Morin et al., 2015) and the observation of reductions in RF RMS activity levels over sprints repetition (Brocherie et al., 2015b), it cannot be ruled out that this finding may partly depend on our non-specific testing position (i.e., marked hip extension while seating). Potentially, different results would occur when adopting another posture (i.e., when lying down) since knee position (RF muscle length) significantly affects quadriceps activation strategies (Krishnan et al., 2011). While this result was unexpected, larger activation in the *soleus* muscle has also been observed +24-h post-football game in hot vs. neutral environment (Girard et al., 2015c). Explosive force production depends on muscle fascicle shortening velocity and the tendon's elastic energy storage capacity, with tendon stiffness in turn affecting the time lag between muscle activation and muscle force production (i.e., electromechanical delay) (Proske and Morgan, 1987). In our study, a fixed electromechanical delay was used to determine EMG onset rise. As such, the role of tension-sensitive mechanoreceptors located in the muscle (e.g., Golgi tendon organs and muscle spindles) in influencing the tendon's stiffness, and thereby length change of the muscle fibers during explosive KE through proprioceptive feedback, probably did not change. A further result was to demonstrate that hypoxia severity had no effect on post-exercise adjustments in rapid muscle activation capacity. Potentially, longer sprints, shorter recoveries or combination of both (more intense exercise-to-rest ratios) and/or the use or severer hypoxic conditions might yield more unfavorable results with respect to RTD adjustments resulting directly to decreases in muscle activation rates if greater fatigue levels could be attained.

### Additional considerations

Importantly, a marked reduction in the intrinsic contractile capacity for explosive force production cannot be ruled out, as substantial peripheral locomotor muscle fatigue development (i.e., twitch torque decrease) usually occur as a result of repeated sprinting, independently of hypoxic severity (Billaut et al., 2013). Assessment of electrically evoked RTD can give insight into the intrinsic capacity of the muscle-tendon unit for explosive force production without the influence of voluntary control. This can be investigated by examining the response to a single or ideally high frequency contractions, such as an evoked octet (e.g., eight pulses at 300 Hz; Buckthorpe et al., 2012), to reliability evoke the maximum capacity for RTD.

In our study, all pre- and post-neuromuscular assessments were performed in normoxia with similar SpO_2_ values of 96–97%. Reportedly, SpO_2_ recovery response after an acute exposure to normobaric hypoxia (FiO_2_ = 10%) decreasing SpO_2_ to 85% is ~2 min (Krivoshchekov et al., 2014). With hypoxic simulation applied during the repeated sprint exercise through the use of a facemask, participants were switched to normoxic breathing immediately (within seconds) after exercise cessation and seated on the chair, located near by the treadmill, for post-test assessment that started exactly 1 min after the last sprint completion. Whether this maneuver induced a faster recovery of neuromuscular function parameters, compared to situations where individuals continued breathing a hypoxic mixture (i.e., similar to exercise conditions), is unknown.

When evaluating RTD, most of the studies have used the same contraction, with a “hard and strong” instruction, to evaluate explosive and maximal voluntary strength capacities. The potential problem associated with this practice, however, is that only ballistic contractions (i.e., force production as fast as possible followed by muscle relaxation as soon the target force is reached) allow a careful evaluation of the maximal discharge rate of motor neurons (Duchateau and Baudry, 2014). Compared with a “hard-and-fast instruction,” the steeper force development with a “fast” only instruction relates to a better activation of the agonist muscles at contraction onset (Sahaly et al., 2003). Although using this methodological precaution required a greater number of contractions (and potentially may have induced some recovery in neuromuscular function), we felt that it was a necessary prerequisite not to underestimate the true rate of muscle activation of our participants.

Because elite players are more accustomed to repeated-sprint activities, one can assume that, in comparison to recreational team-sport participants involved here, they may have been able to better resist fatigue. In a group of 17 healthy recreationally active individuals, those facing the greatest RTD reductions also experienced the largest fatigue rate during a Wingate cycle ergometer test and greater fatigue during an electrical stimulation protocol (Morris et al., 2010). Along the same line, only males were studied here, while females are generally less fatigable compared with men during repeated-sprint protocols (Billaut and Bishop, 2009). Whether performance level and/or gender differences exist regarding neuromuscular consequences (with special references to explosive strength), when completing repeated-sprint exercises at different altitudes, warrant further investigation.

## Conclusion

In summary, alteration in repeated-sprint ability and post- KE MVC was greater under high altitude than in normoxia or at low altitude. In the KE, peak and late phase (>100 ms) contractile RTD decreased post-exercise to the same extent between conditions. However, contractile RTDs were not different after normalization to MVC torque, indicating that post-exercise strength losses accounted for the decrease in RTD. Additionally, we reported that repeated running sprints do not negatively influence the capacity of the central nervous system to rapidly activate the VL (unchanged) and RF (improved) muscles during the first 200 ms, whereas maximal activation was dampened later during the contraction. Finally, normobaric hypoxia exposure had no additional influence on post-exercise alterations in rapid muscle torque production of the KE.

## Author contributions

Conceived and designed the experiments: OG, FB, GM. Performed experiments: OG, FB. Analyzed data: OG. Interpreted results of research: OG. Drafted manuscript and prepared tables/figures: OG. Edited, critically revised paper, and approved final version of manuscript: OG, FB, GM.

## Funding

This work is based on research funded by QNRF (NPRP 4–760–3–217).

### Conflict of interest statement

The authors declare that the research was conducted in the absence of any commercial or financial relationships that could be construed as a potential conflict of interest.

## References

[B1] Aagaard P. Simonsen E. B. Andersen J. L. Magnusson P. Dyhre-Poulsen P. (2002). Increased rate of force development and neural drive of human skeletal muscle following resistance training. J. Appl. Physiol. 93, 1318–1326. 10.1152/japplphysiol.00283.2002 12235031

[B2] Andersen L. L. Aagaard P. (2006). Influence of maximal muscle strength and intrinsic muscle contractile properties on contractile rate of force development. Eur. J. Appl. Physiol. 96, 46–52. 10.1007/s00421-005-0070-z 16249918

[B3] Andersen L. L. Andersen J. L. Zebis M. K. Aagaard P. (2010). Early and late rate of force development: differential adaptive responses to resistance training? Scand. J. Med. Sci. Sports 20, 162–169. 10.1111/j.1600-0838.2009.00933.x 19793220

[B4] Balsom P. D. Gaitanos G. C. Ekblom B. Sjödin B. (1994). Reduced oxygen availability during high intensity intermittent exercise impairs performance. Acta Physiol. Scand. 152, 279–285. 10.1111/j.1748-1716.1994.tb09807.x 7872005

[B5] Billaut F. Bishop D. J. (2009). Muscle fatigue in males and females during multiple-sprint exercise. Sports Med. 39, 257–278. 10.2165/00007256-200939040-00001 19317516

[B6] Billaut F. Kerris J. P. Rodriguez R. F. Martin D. T. Gore C. J. Bishop D. J. (2013). Interactions of central and peripheral factors using repeated sprints at different levels of arterial O_2_ saturation. PLoS ONE 8:e77297. 10.1371/journal.pone.0077297 24155938 PMC3796493

[B7] Bishop D. J. Girard O. Mendez-Villanueva A. (2011). Repeated-sprint ability – Part II. Recommendations for training. Sports Med. 49, 741–756. 10.2165/11590560-000000000-00000 21846163

[B8] Bowtell J. L. Cooke K. Turner R. Mileva K. N. Sumners D. P. (2014). Acute physiological and performance responses to repeated sprints in varying degrees of hypoxia. J. Sci. Med. Sport 17, 399–403. 10.1016/j.jsams.2013.05.016 23809839

[B9] Brocherie F. Girard O. Faiss R. Millet G. P. (2015a). High-intensity intermittent training in hypoxia: a double-blinded, placebo-controlled field study in youth football players. J. Strength Cond. Res. 29, 226–237. 10.1519/JSC.0000000000000590 24978836

[B10] Brocherie F. Millet G. P. Girard O. (2015b). Neuro-mechanical and metabolic adjustments to the repeated anaerobic sprint test in professional football players. Eur. J. Appl. Physiol. 115, 891–903. 10.1007/s00421-014-3070-z 25481506

[B11] Buckthorpe M. W. Hannah R. Pain T. G. Folland J. P. (2012). Explosive isometric contractions, with special reference to electromyography normalization techniques. Muscle Nerve 46, 566–576. 10.1002/mus.23322 22987699

[B12] Buckthorpe M. Pain M. T. Folland J. (2014). Central fatigue contributes to the greater reductions in explosive than maximal strength with high-intensity fatigue. Exp. Physiol. 99, 964–973. 10.1113/expphysiol.2013.075614 24728678

[B13] Calbet J. A. De Paz J. A. Garatechea N. Cabeza de Veca S. Chavarren J. (2003). Anaerobic energy provision does not limit Wingate exercise performance in endurance-trained cyclists. J Appl Physiol. 94, 668–676. 10.1152/japplphysiol.00128.2002 12391104

[B14] Christian R. J. Bishop D. J. Billaut F. Girard O. (2014). The role of sense of effort on self-selected cycling power output. Front. Physiol. 5:115. 10.3389/fphys.2014.00115 24744734 PMC3978313

[B15] de Oliveira F. B. Rizatto G. F. Denadai B. S. (2013). Are early and late force development differently influenced by fast-velocity resistance training? Clin. Physiol. Funct. Imaging 33, 282–287. 10.1111/cpf.12025 23692617

[B16] Duchateau J. Baudry S. (2014). Maximal discharge rate of motor units determines the maximal rate of force development during ballistic contractions in human. Front. Hum. Neurosci. 8:234. 10.3389/fnhum.2014.00234 24795599 PMC4001023

[B17] Edman K. A. Josephson R. K. (2007). Determinants of force rise time during isometric contraction of frog muscle fibres. J. Physiol. 580, 1007–1019. 10.1113/jphysiol.2006.119982 17303645 PMC2075450

[B18] Feriche B. García-Ramos A. Calderón-Soto C. Drobnic F. Bonitch-Gongóra J. G. Galilea P. A.. (2014). Effect of acute exposure to moderate altitude on muscle power: hypobaric hypoxia vs. normobaric hypoxia. PLoS ONE 9:e114072. 10.1371/journal.pone.0114072 25474104 PMC4256399

[B19] Folland J. P. Buckthorpe M. W. Hannah R. (2014). Human capacity for explosive force production: neural and contractile determinants. Scand. J. Med. Sci. Sports 24, 894–906. 10.1111/sms.12131 25754620

[B20] Gandevia S. C. (2001). Spinal and supraspinal factors in human muscle fatigue. Physiol. Rev. 81, 1725–1789. 11581501 10.1152/physrev.2001.81.4.1725

[B21] Girard O. Bishop D. J. Racinais S. (2013). Hot conditions improve power output during repeated cycling sprints without modifying neuromuscular fatigue characteristics. Eur. J. Appl. Physiol. 113, 359–369. 10.1007/s00421-012-2444-3 22743981

[B22] Girard O. Brocherie F. Morin J. B. Millet G. P. (2015a). Neuro-mechanical determinants of repeated treadmill sprints - Usefulness of an “hypoxic to normoxic recovery” approach. Front. Physiol. 6:260 10.3389/fphys.2015.00260 PMC4585155 26441679

[B23] Girard O. Brocherie F. Morin J. B. Millet G. P. (2015b). Intra- and inter-session reliability of running mechanics during treadmill sprints. Int. J. Sports Physiol. Perform. [Epub ahead of print]. 10.1123/ijspp.2015-0145 26356384

[B24] Girard O. Mendez-Villanueva A. Bishop D. J. (2011). Repeated-sprint ability-Part I. Sports Med. 41, 673–694. 10.2165/11590560-000000000-00000 21780851

[B25] Girard O. Millet G. (2009). Maximal rate of force development can represent a more functional measure of muscle activation. J. Appl. Physiol. 107, 359–360. 19670469 10.1152/japplphysiol.00362.2009

[B26] Girard O. Nybo L. Mohr M. Racinais S. (2015c). Plantar flexor neuromuscular adjustments following match-play football in hot and cool conditions. Scand. J. Med. Sci. Sports 25, 154–163. 10.1111/sms.12371 25943666

[B27] Girard O. Racinais S. Périard J. (2014). Tennis in hot and cool conditions decreases the rapid muscle torque production capacity of the knee extensors but not the plantar flexors. Br. J. Sports Med. 48, 52–58. 10.1136/bjsports-2013-093286 PMC3995226 24668381

[B28] Goodall S. Charlton K. Howatson G. Thomas K. (2015). Neuromuscular fatigability during repeated-sprint exercise in male athletes. Med. Sci. Sports Exerc. 47, 528–536. 10.1249/MSS.0000000000000443 25010404

[B29] Goodall S. Ross E. Z. Romer L. M. (2010). Effect of graded hypoxia on supraspinal contributions to fatigue with unilateral knee-extensor contractions. J. Appl. Physiol. 109, 1842–1851. 10.1152/japplphysiol.00458.2010 20813979

[B30] Goods P. S. R. Dawson B. T. Landers G. J. Gore C. J. Peeling P. (2014). Effect of different simulated altitudes on repeat-sprint performance in team-sport athletes. Int. J. Sports Physiol. Perform. 9, 857–862. 10.1123/ijspp.2013-0423 24509626

[B31] Griffin L. Cafarelli E. (2005). Resistance training: cortical, spinal, and motor unit adaptations. Can. J. Appl. Physiol. 30, 328–340. 10.1139/h05-125 16129897

[B32] Hamada T. Sale D. G. MacDougall J. D. Tarnopolsky M. A. (2003). Interaction of fibre type, potentiation and fatigue in human knee extensor muscles. Acta Physiol. Scand. 178, 165–173. 10.1046/j.1365-201X.2003.01121.x 12780391

[B33] Harridge S. D. Bottinelli R. Canepari M. Pellegrino M. A. Reggiani C. Esbjornsson M.. (1996). Whole-muscle and single-fibre contractile properties and myosin heavy chain isoforms in humans. Pflugers Arch. 432, 913–920. 10.1007/s004240050215 8772143

[B34] Holtermann A. Roeleveld K. Vereijken B. Ettema G. (2007). The effect of rate of force development on maximal force production: acute and training-related aspects. Eur. J. Appl. Physiol. 99, 605–613. 10.1007/s00421-006-0380-9 17219170

[B35] Hureau T. J. Olivier N. Millet G. Y. Meste O. Blain G. M. (2014). Exercise performance is regulated during repeated sprints to limit the development of peripheral fatigue beyond a critical threshold. Exp. Physiol. 99, 951–963. 10.1113/expphysiol.2014.077974 24728680

[B36] Krishnan C. Allen E. J. Williams G. N. (2011). Effect of knee position on quadriceps muscle force steadiness and activation strategies. Muscle Nerve. 43, 563–573. 10.1002/mus.21981 21404288 PMC3077092

[B37] Krivoshchekov S. G. Balioz N. V. Nekipelova N. V. Kapilevich L. V. (2014). Age, gender, and individually-typological features of reaction to sharp hypoxic influence. Hum. Physiol. 40, 34–45. 10.1134/S0362119714060061 25711107

[B38] Kuitunen S. Komi P. V. Kyröläinen H. (2002). Knee and ankle joint stiffness in sprint running. Med. Sci. Sports Exerc. 34, 166–173. 10.1097/00005768-200201000-00025 11782663

[B39] Levine B. D. Stray-Gundersen J. Mehta R. D. (2008). Effect of altitude on football performance. Scand. J. Med. Sci. Sports 18, 76–84. 10.1111/j.1600-0838.2008.00835.x 18665955

[B40] Luhtanen P. Komi P. V. (1979). Mechanical power and segmental contribution to force impulses in long jump take-off. Eur. J. Appl. Physiol. Occup. Physiol. 41, 267–274. 10.1007/BF00429743 499190

[B41] Millet G. P. Faiss R. Pialoux V. (2012). Point: hypobaric hypoxia induces different physiological responses from normobaric hypoxia. J. Appl. Physiol. 112, 1783–1784. 10.1152/japplphysiol.00067.2012 22267386

[B42] Morel B. Rouffet D. M. Saboul D. Rota S. Clémençon M. Hautier C. (2015). Peak torque and rate of torque development influence on repeated maximal exercise performance: contractile and neural contributions. PLoS ONE 10:e0119719. 10.1371/journal.pone.0119719 25901576 PMC4406491

[B43] Morin J. B. Gimenez P. Edouard P. Arnal P. Jiménez-Reyes P. Samozino P.. (2015). Sprint acceleration mechanics: the major role of hamstrings in horizontal force production. Front. Physiol. 6:404. 10.3389/fphys.2015.00404 26733889 PMC4689850

[B44] Morin J. B. Samozino P. Bonnefoy R. Edouard P. Belli A. (2010). Direct measurement of power during one single sprint on treadmill. J. Biomech. 43, 1970–1975. 10.1016/j.jbiomech.2010.03.012 20541762

[B45] Morris M. G. Dawes H. Howells K. Scott O. M. Cramp M. Izadi H. (2010). Muscle contractile characteristics: relationship to high-intensity exercise. Eur. J. Appl. Physiol. 110, 295–300. 10.1007/s00421-010-1496-5 20467873

[B46] Nelson A. G. (1996). Supramaximal activation increases motor unit velocity of unloading. J. Appl. Biomech. 12, 285–291.

[B47] Pearcey G. E. Murphy J. R. Behm D. G. Hay D. C. Power K. E. Button D. C. (2015). Neuromuscular fatigue of the knee extensors during repeated maximal intensity intermittent-sprints on a cycle ergometer. Muscle Nerve 51, 569–579. 10.1002/mus.24342 25043506

[B48] Penailillo L. Blazevich A. Numazawa H. Nosaka K. (2014). Rate of force development as a measure of muscle damage. Scand. J. Med. Sci. Sports 3, 417–427. 10.1111/sms.12241 24798498

[B49] Perrey S. Racinais S. Saimouaa K. Girard O. (2010). Neural and muscular adjustments following repeated running sprints. Eur. J. Appl. Physiol. 109, 1027–1036. 10.1007/s00421-010-1445-3 20358218

[B50] Perrey S. Rupp T. (2009). Altitude-induced changes in muscle contractile properties. High Alt. Med. Biol. 10, 175–182. 10.1089/ham.2008.1093 19519224

[B51] Proske U. Morgan D. L. (1987). Tendon stiffness: methods of measurement and significance for the control of movement. A review. J. Biomech. 20, 75–82. 10.1016/0021-9290(87)90269-7 3558432

[B52] Racinais S. Bishop D. Denis R. Lattier G. Mendez-Villaneuva A. Perrey S. (2007). Muscle deoxygenation and neural drive to the muscle during repeated sprint cycling. Med. Sci. Sports Exerc. 39, 268–274. 10.1249/01.mss.0000251775.46460.cb 17277590

[B53] Racinais S. Maffiuletti N. A. Girard O. (2013). M-wave, H- and V-Reflex recruitment curves during maximal voluntary contraction. J. Clin. Neurophysiol. 30, 415–421. 10.1097/WNP.0b013e31829ddcf1 23912583

[B54] Sahaly R. Vandewalle H. Driss T. Monod H. (2003). Surface electromyograms of agonist and antagonist muscle during force development of maximal isometric exercises - effects of instruction. Eur. J. Appl. Physiol. 89, 79–84. 10.1007/s00421-002-0762-6 12627309

[B55] Spencer M. Bishop D. Dawson B. Goodman C. (2005). Physiological and metabolic responses of repeated-sprint activities: specific to field-based team sports. Sports Med. 35, 1025–1044. 10.2165/00007256-200535120-00003 16336007

[B56] Suetta C. Aagaard P. Rosted A. Jakobsen A. K. Duus B. Kjaer M.. (2004). Training-induced changes in muscle CSA, muscle strength, EMG and rate of force development in elderly subjects after long-term unilateral disuse. J. Appl. Physiol. 97, 1954–1961. 10.1152/japplphysiol.01307.2003 15247162

[B57] Thorlund J. B. Aagaard P. Madsen K. (2009). Rapid muscle force capacity changes after soccer match play. Int. J. Sports Med. 30, 273–278. 10.1055/s-0028-1104587 19199196

[B58] Thorlund J. B. Michalsik L. B. Madsen K. Aagaard P. (2008). Acute fatigue-induced changes in muscle mechanical properties and neuromuscular activity in elite handball players following a handball match. Scand. J. Med. Sci. Sports 18, 462–472. 10.1111/j.1600-0838.2007.00710.x 18028284

[B59] Thorstensson A. Karlsson J. Viitasalo J. H. Luhtanen P. Komi P. V. (1976). Effect of strength training on EMG of human skeletal muscle. Acta Physiol. Scand. 98, 232–236. 10.1111/j.1748-1716.1976.tb00241.x 983733

[B60] Tillin N. A. Pain M. T. Folland J. (2013). Explosive force production during isometric squats correlates with athletic performance in rugby union players. J. Sports Sci. 31, 66–76. 10.1080/02640414.2012.720704 22938509

